# A Nanostructured Piezoelectric Immunosensor for Detection of Human Cardiac Troponin T

**DOI:** 10.3390/s111110785

**Published:** 2011-11-16

**Authors:** Rosana A. S. Fonseca, Joilson Ramos-Jesus, Lauro T. Kubota, Rosa F. Dutra

**Affiliations:** 1 Laboratório de Engenharia Biomédica, Universidade Federal de Pernambuco, Av. Prof. Moraes Rego, 1235, Recife, Pernambuco 50670-901, Brazil; E-Mails: rosanafonseca@hotmail.com (R.A.S.F.); joiramos@hotmail.com (J.R.-J.); 2 Instituto de Química, Universidade Estadual de Campinas, Campinas 04118-080, SP, Brazil; E-Mail: kubota@iqm.unicamp.br

**Keywords:** piezoelectric, immunosensor, gold nanoparticle, cardiac troponin T

## Abstract

A piezoelectric immunosensor based on gold nanoparticles (AuNPs) co-immobilized on a dithiol-modified surface is proposed for detection of human cardiac troponin T (TnT). Anti-human troponin T (anti-TnT) antibodies were covalently immobilized on the nanostructured electrode surface by thiol-aldehyde linkages. In a homogeneous bulk solution, TnT was captured by anti-TnT immobilized on the QCM electrode. Cyclic voltammetry studies were used to characterize the AuNPs layer on the electrode surface and the anti-TnT immobilization steps. The QCM-flow immunosensor exhibited good reliability, measuring concentrations of TnT from 0.003 to 0.5 ng mL^−1^ in human serum with high linearity (r = 0.989; p < 0.01). The immunosensor exhibited a 7% coefficient of variation and 0.0015 ng mL^−1^ limit of detection, indicating a high reproducibility and sensitivity. The proposed QCM nanostructured immunosensor is easy to use and has promising potential in the diagnosis of acute myocardial infarction due to its speed and high sensitivity.

## 1. Introduction

Cardiovascular disease accounts for nearly half of all deaths in the European Union [1,2] and approximately one third of deaths in Brazil [3]. The timely, accurate diagnosis of acute coronary syndromes, such as acute myocardial infarction (the most serious manifestation of coronary ischemia), is essential. In the 1990s, the cardiac troponin assay revolutionized the diagnostics for patients presenting with acute coronary syndrome, as increased levels of troponin T (TnT) are highly specific to cardiac injury, correlate well with the extent of myocardial necrosis and are strongly associated with an increased risk of reinfarction and death [4,5]. TnT is a 30–35-kDa protein ranging in size from 223 to 305 amino acids [6,7] and is expressed exclusively in cardiac myocytes. The TnT concentration surpasses the 0.3 ng mL^−1^ threshold in the circulation 3 to 4 h after the onset of myocardial injury.

Complications after acute myocardial infarction reach a maximum in the first few hours and decrease with the passing of time. Early diagnosis is important to planning treatment modalities, such as thrombolytic therapy, coronary artery bypass graft and other therapeutic interventions, which, if instituted in time, considerably reduce morbidity and mortality rates. Thus, the development of quantitative methods for the determination of TnT using a bedside serum troponin-T test is highly desirable. Electrochemiluminescence immunoassay (ECLIA) and enzyme-linked immunosorbent assay (ELISA) are the current methods for detecting troponin. However, these methods are not practical and require samples to be processed at a central location [8–10]. The ELISA test consists of a sandwich-type, which are used in two anti-TnT, one being marked with the peroxidase enzyme. This test is performed in two steps and takes approximately 60 min to perform. The ECLIA method is based on a single step sandwich principle, with strepavidin-coated tubes to the solid phase and two monoclonal human cardiac anti- troponinT. One of these antibodies is marked with rubidium, an atom emits luminescence. This test takes some 20 min [10]. In recent years, biosensors have emerged as an attractive alternative for overcoming these difficulties.

Different types of transducers have been explored for constructing piezoelectric sensors in an attempt to minimize response time and enable miniaturization. Such systems are based on the frequency change caused by the amount of mass adsorbed on the quartz crystal electrode surface coupled to an oscillator circuit. In the immunossensor approach, the change in mass is caused when a biological adsorbent material (antigen) interacts with its complementary species on the electrode surface (antibody), thereby increasing the dielectric strength of the crystal and consequently reducing the oscillation of electric frequency [11]. The relationship between the frequency shift (Δf) of the quartz crystal resonator and changes in its mass per unit of surface area (Δm) is described by the Sauerbrey equation (Equation (1)) [12]:

(1) $$\Delta f=-\frac{2f_{0}^{2}}{A{\left(\mu_{q}\rho_{q}\right)}^{1/2}}\Delta m$$

where *μ*_q_ and *ρ*_q_ are the shear modulus and density of the quartz (2.95 × 10^11^ dyne/cm^2^ and 2.65 g/cm^3^, respectively), *f*_0_ is the fundamental oscillation frequency of the dry crystal and Δ*m* is the mass of the material adsorbed on the surface per unit/area.

The immobilization of biomolecules such as antibodies on a gold electrode surface is a crucial step in the development of immunosensors. Immobilization strategies must maintain the integrity and bioreactivity of the immobilized molecules [13,14]. There are different immobilization methods employing sulfurated organic compounds for attaching biomolecules on gold surfaces, such as amino-alkanethiols, thioacids and alkanedithiols. The use of self-assembled monolayers (SAMs) for the immobilization of active biomolecules has led to immunosensors with satisfactory performance [15,16]. SAMs are molecular structures formed from suitable substrates such as alkanethiols that spontaneously self-organize on an adequate metal surface on a nanometric scale as sulfur is strongly attracted to the gold surface [17,18].

SAMs and nanomaterials are promising structures for improving the effective, stable immobilization of biomolecules on sensor platforms, especially when high sensitivity is required. Among these nanomaterials, gold nanoparticles (AuNPs) have been widely used to enhance the sensitivity of the devices due to increase the surface area of an electrode [19–21]. In recent years, a number of methods have been developed for the construction of thin polymer films containing AuNPs on Au surfaces. However, this type of immobilization had the disadvantage of losing sensitivity rapidly with time [22]. The use of small immobilized AuNPs, with a SAM to immobilize proteins on a surface of the electrode improves the response of the electrode, because these nanoparticles to allow more freedom to antibodies or antigens anchored [23]. Here, AuNPs were attached to the surface of the quartz crystal through the 1,6-hexanedithiol linker (HDT), allowing greater exposure of the surface of the AuNPs. This nanostructure layer allows covalent bonding of a larger amount of antibodies or antigens [24,25]. The present study proposes a piezoelectric immunosensor for the detection of human cardiac troponin T (TnT) based on a AuNPs monolayer co-immobilized on a dithiol-modified surface. For this purpose, anti-TnT was immobilized on the nanostructured electrode surface through cystamine, which acted as a cross-linking spacer.

## 2. Experimental Section

### 2.1. Apparatus

Quartz crystals (9 MHz) coated with a thin layer of chrome covered with polished gold on both sides (Maxtek Inc., Cypress, CA, USA) were used. The thickness of the quartz, chrome and gold layers was 0.2 mm, 20 nm and 50 nm, respectively, and the diameter of these layers was 12.5, 6 and 6 mm, respectively. The piezoelectric crystal was placed on a cell with two gold terminals 6 mm in diameter. The cell was covered on the upper and lower sides by two polystyrene rings. The quartz crystal microbalance (QCM) was closed with a plastic cap containing two metal pins to which tubes were attached to allow continuous flow cleaning of the crystal when a peristaltic pump was switched on (SJ 1211H, series 253512, Chromatograph Atta, Tokyo, Japan). QCM and an AUTOLAB potentiostat (Eco Chemie, Utrecht, The Netherlands) were used to form a high-resolution integrated oscillating circuit. The modular set of equipment was linked to a computer to record the measurements.

The characterization of the surface coated with gold nanoparticles was performed using an electrochemical technique involving cyclic voltammetry. Electric current measurements were performed at a constant electric potential and scan rate during the immobilization of anti-TnT.

### 2.2. Reagents

1,6-hexanedithiol (HDT), colloidal gold (5 nm) in a hydrochloric medium, cystamine (CYS), glutaraldehyde (GLU) and glycine (GLY) were acquired from Sigma-Aldrich (St. Louis, MO, USA). The monoclonal biotinylated anti-TnT antibody (200 μg mL^−1^) and human cardiac troponin T (10 ng mL^−1^, lot H0505) were acquired from Roche Diagnostics (Mannheim, Germany). All other reagents used in the present study were of analytical grade purity. Phosphate buffer solutions (PBS) with different pH values and in different concentrations were prepared using adequate of Na_2_HPO_4_, KH_2_PO_4_, NaCl and KCl. In the anti-TnT concentration studies, 10 mmol L^−1^ PBS was used, dissolving 0.24 g of Na_2_HPO_4_, 1.44 g of KH_2_PO_4_, 0.2 g of KCl, 8.0 g of NaCl in 1,000 mL of ultra-pure water, with the pH adjusted to 7.4. “Piranha” solution used for cleaning the electrode consisted of a 5:1 mixture of concentrated sulfuric acid to 30% hydrogen peroxide solution, which was diluted 1:5 in ultra-pure Milli-Q water. Due to the self-decomposition of hydrogen peroxide, this mixture was always used freshly prepared.

### 2.3. Serum Samples

A pool of human serum samples was obtained from 10 healthy volunteers (five males and five females). For all serum sample collections, the blood was collected in 10-mL vacutainer serum tubes (BD, Franklin Lakes, NJ, USA), allowed to stand for 15 min and then centrifuged at 3,500 g for 25 min. The serum was transferred from the vacutainer tubes into polypropylene tubes and stored at −20 °C until analysis. This pooled serum was established as control. The amount of TnT was measured using ECLIA (Elecsys analyser 1010, Roche), which presented 0.0015 ng mL^−1^ TnT. Serum samples with different concentrations of TnT for the evaluation of immunosensor response were obtained by adding a known amount of TnT to the pooled serum.

### 2.4. Immobilization of Anti-TnT on QCM Electrode

The gold surface of the piezoelectric crystal was cleaned twice with the “piranha” solution for 5 min and rinsed with ultra-pure distilled water. After the chemical cleaning, successive washes with distilled water followed by PBS were performed using a peristaltic pump for 10 min at a speed of 700 μL min^−1^. The piezoelectric crystal was placed on the detection cell and sealed with an o-ring, leaving only one side of the gold electrode in contact with the solution for reactions. The surface of the crystal electrode was then incubated with a solution of 20 mmol L^−1^ of HDT prepared in ethanol for 2 h with the flow stopped. After washing in PBS, the gold electrode was incubated for 14 h in a solution of AuNP (1 ng mL^−1^) prepared in PBS (pH 9.0, 10 mmol L^−1^). The nanostructured gold electrode surface was incubated with ethanol solution of 25 mM CYS (H_12_N_2_S_2_·2HCl) for 2 h. Linkage of the anti-TnT was then performed through aldehyde groups by incubating with the modified surface with 2.5% GLU solution in PBS for 45 min. Finally, an anti-TnT solution (150 ng mL^−1^) was incubated on the functionalized nanostructured gold electrode surface for 2 h. Non-specific bindings were blocked by the use of 50 mmol L^−1^ of glycine solution for 2 h. Between all incubations step, the crystal was washed with a continuous flow of PBS for 10 min.

### 2.5. Electrochemical Characterization of Electrode Surface

Cyclic voltammetry studies were carried out using an electrode system composed of an anti-TnT/AuNP/HDT/Au electrode as the working electrode, Ag/AgCl as the reference electrode and Pt wire as the auxiliary electrode. To discern the role of the individual components, cyclic voltammograms of the bare Au electrode, HDT/Au electrode, AuNP/HDT/Au and Gly/Anti-TnT/AuNP/HDT/Au electrode were recorded in 25 mmol L^−1^ PBS (pH 7.2) containing 0.1 M K_3_Fe(CN)_6_^3/4−^ in aqueous KCl as the redox probe at a scan rate of 0.0 to +1 mV s^−1^ at an interval of 50 mV s^−1^.

## 3. Results and Discussion

### 3.1. Characterization of Anti-TnT Immobilization

Cyclic voltammetry is commonly used to monitor the process of biomolecule immobilization. Cyclic voltammograms were recorded using 10 mmol L^−1^ K_3_Fe(CN)_6_^3/4−^ in 0.1 M aqueous KCl for the redox probe. To ensure a good Au-S bond, the electrode surface should be properly cleaned [26]. The gold electrode was pretreated with a “piranha” solution in order to clean the surface and form gold oxide, which facilitates the linkage with -SH groups from HDT [Equation (2)]:

(2) $$\text{Au}+\text{HS}-{\left({\text{CH}}_{2}\right)}_{6}-\text{SH}\;\to \;\text{Au}-S-{\left({\text{CH}}_{2}\right)}_{6}-\text{SH}+½H_{2}$$

**Figure 1.**
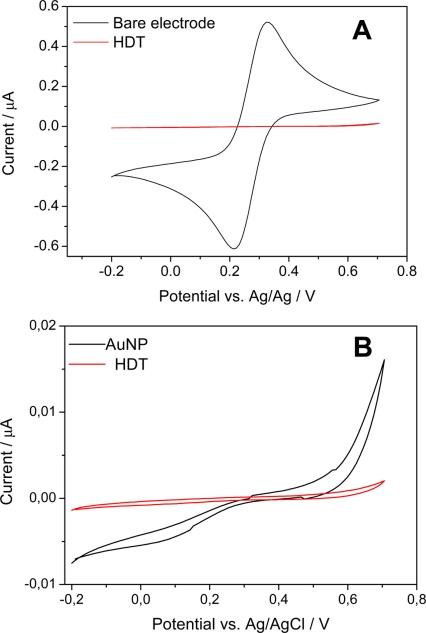

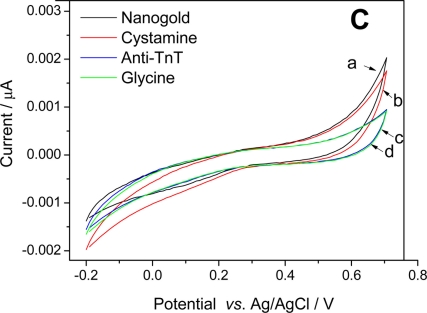
Cyclic voltammograms of modified AuNP/CYS/Anti-TnT/GLY electrode; **(A)** bare and modified Au electrode with HDT; **(B)** modified electrode with HDT and AuNPs; **(C)** after absorption of AuNPs, CYS + GLU, anti-TnT immobilization and GLY blocking corresponding to a, b, c and d, respectively.

To obtain greater effectiveness in the linkage of the thiol groups on the gold electrode, the surface was pre-polarized to 0.7 V potential *vs*. Ag/AgCl during 4 min. The proximity of the peak potential of oxidation and reduction exhibited by Fe(CN)_6_^3−/4−^ redox probe of the bare gold electrode with a difference less than 0.1 V [Figure 1(A)] is an indicative of the fact that the cleaning procedure was effective. Considerable attention has been focused on dithiol films on gold electrodes [27]. Compared to the response of the bare gold electrode, a substantial reduction of the redox peaks was observed. This behavior is attributed to film deposition on the electrode surface through HDT. An increased oxidation current compared to the reduction current reinforced by a reductive desorption of these monolayers in thiol according to Equation (3) was also observed [28]:

(3) $$\text{RS}-\text{Au}+e^{-}\to \text{Au}+{\text{RS}}^{-}$$

Figure 1(B) displays cyclic voltammograms for a gold electrode in HDT and AuNPs solutions. The HDT-modified electrode exhibited significant oxidation and reduction currents starting at around 0.7 and −0.2 V, respectively, indicating a fast follow-up chemical reaction [27]. The AuNP-modified gold electrode exhibited a very weak electrochemical response over this potential range as well a decrease in the area of the voltammogram, suggesting the formation of a blocking film on the electrode surface, likely due to the addition of AuNPs negative charges which are linked to the QCM electrode surface by the HDT that increased conductivity on the electrostatic electrode surface [28,29].

Although a remarkable reduction wave is observed for the AuNP electrode in comparison to HDT, the AuNP electrode exhibited (a) a larger anodic current than the (b) cystamine, (c) anti-TnT and (d) glycine electrodes, indicating that the nano-gold-HDT connection was successful. The overlapping areas of the voltammograms for anti-TnT and glycine reveal that nanogold-cystamine immobilization allowed the adequate coating of the electrode surface [Figure 1(C)]. The formation of SAMs building on gold surfaces allows control of the amount and orientation of the antibodies. However, the use of planar gold as the transducing surface may lead to a limited number of accessible antibodies [30]. Gold nanoparticles have been successfully used to enhance the sensitivity of biomolecular detection assays by improving antibody immobilization, as AuNPs increase the surface contact of the electrode, which leads to a corresponding increase in the number of immobilized antibodies per unit of area on the electrode.

### 3.2. Anti-TnT Immobilization

**Figure 2.**
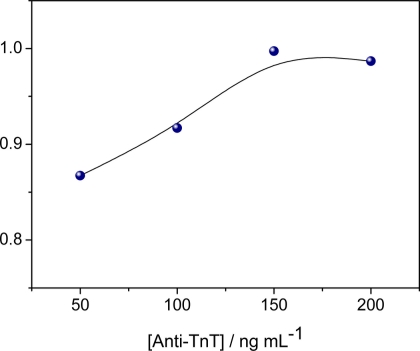
Dependence of anti-TnT concentration on immunosensor sensitivity.

The effects of the anti-TnT concentration immobilized on the quartz electrode were investigated in relation to the maximum sensitivity of the immunosensor. Solutions of different concentrations of anti-TnT prepared in PBS (50, 100, 150 and 200 ng mL^−1^) were immobilized on the nanostructured electrode and the optimal anti-TnT concentration was established through the maximal response to conjugation with the TnT antigen suspended in PBS (pH 7.4, 10 mmol L^−1^). Figure 2 displays the regression correlation “r” values obtained from calibration curves with different concentrations of immobilized anti-TnT. Maximal equilibrium between antibody and antigen was achieved at 150 ng mL^−1^ anti-TnT, as evident by the plateau reached, which may be attributed to an equivalence zone. Thus, the TnT concentration of 150 ng mL^−1^ was used in all remaining experiments.

### 3.3. Effect of Ionic Strength on Sensitivity of Nanostructured Immunosensor

**Figure 3.**
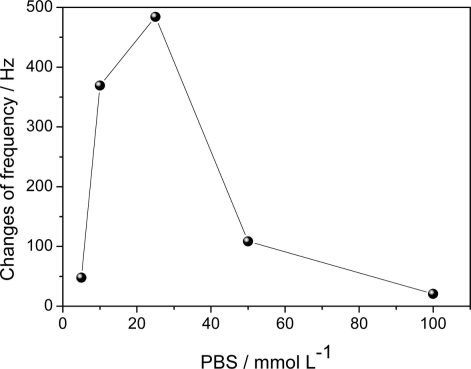
Effect of PBS solution on sensitivity of immunosensor; dependence of PBS concentration on adsorption of anti-TnT to AuNP/HDT/Au nanostructure.

The influence of ionic strength on the immobilization of anti-TnT was investigated (Figure 3). There was an increase in frequency variation when the concentration of PBS increased from 5 mmol L^−1^ to 25 mmol L^−1^, whereas a concentration above 25 mmol L^−1^ led to a reduction in frequency variation. It may be that the concentration of 25 mmol L^−1^ during the immunoreaction promoted greater recognition of the TnT antigen by the immobilized antibody.

### 3.4. Effect of pH on Response of Immunosensor

**Figure 4.**
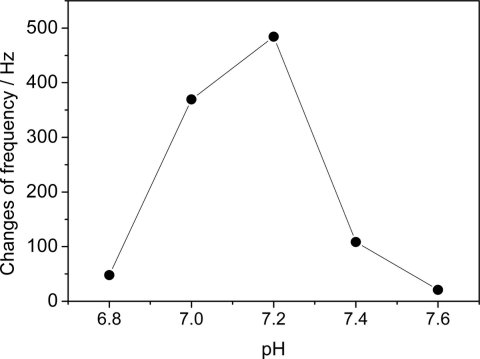
Influence of pH on TnT response in each 1 ng mL^−1^ of TnT solution was prepared in 10 mmol L^−1^ of PBS with different pH values and incubated for 15 min at room temperature; anti-TnT concentration was 150 ng mL^−1^, prepared in 10 mmol L^−1^ of PBS at pH 7.4 and immobilized for 2.5 h at room temperature; frequency measurements performed on QCM.

The piezoelectric quartz crystal was used to study the influence of pH on the response of the immunosensor. The TnT antigen was incubated at a concentration of 1 ng mL^−1^ and suspended in 10 mmols L^−1^ of PBS at different pH values (Figure 4).

The choice of the 6.8 to 7.6 range was made taking into account the possible changes in blood pH that could occur in patients whose condition may result in acidosis or alkalosis (reduction or increase in plasma pH). This variation in blood pH is seen in conditions in which the organism loses its capacity to balance the input of inspired oxygen with the carbon dioxide gas to be eliminated. Moreover, as the pH of the medium decreases, it approaches the isoelectric point of TnT and this fails to interact with the anti-TnT [31]. Figure 4 displays a rise in the frequency variation when the pH of the medium went from 6.8 to 7.2 and a subsequent decline in the curve until a pH value close to 7.5. The greatest frequency variation measured on the QCM was obtained in PBS solution with pH near 7.2, suggesting that this is the optimal pH value, likely due to the fact that this value is less favorable to electrostatic repulsion during immunoreactivity [24]. Thus, pH 7.2 for the PBS solution was established as optimal for the formation of the anti-TnT and TnT antigen complex.

### 3.5. Immunosensor Response to TnT in the Human Serum

**Figure 5.**
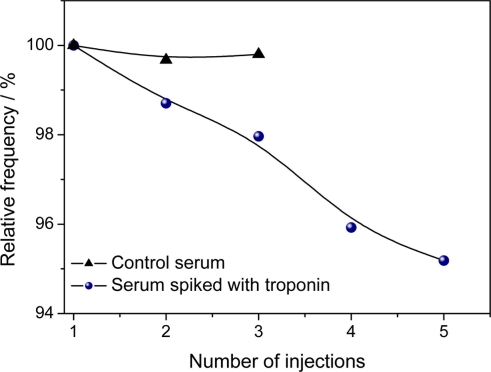
Relative frequency change from the control and serum spiked with troponin. Frequency measurements taken after 15 min of incubation.

The performance of the immunosensor was studied using human serum samples spiked with troponin. The sera were 1:10 diluted in PBS in order to obtain a final concentration of 1 ng/mL TnT. Figure 5 displays a higher slope for TnT serum samples spiked with TnT than control samples indicating a high discrimination of the cardiac TnT in the real samples.

**Figure 6.**
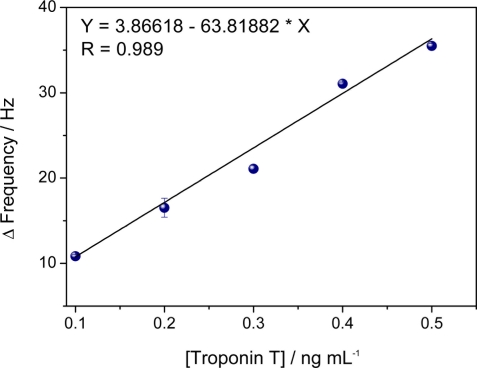
Calibration curve obtained with serum; frequency measurements taken after 15 min of incubation for processing of immunoreaction; reaction conditions based on best results of studies of anti-TnT concentration, PBS concentration and pH of immobilization and immunoreactions.

The sensitivity of the immunosensor was assessed by a test involving the control serum pooled from the volunteers, with the addition of 0.1 ng L^−1^ of TnT solution prepared in PBS (pH 7.2, 25 mmol L^−1^). The measuring procedure for TnT was in flow. During the injection and incubation, the flow was stopped. The measurement of the frequency was performed in duplicate (Figure 6). Serum is a complex medium that contains innumerable biochemical substances such as proteins, enzymes, amino acids, carbohydrates, lipids and ions. Non specific binding by adsorption of these substances may lead to false positive responses by frequency changes. Then, different serum samples were used to evaluate the selectivity of the immunosensor. The immunosensor displayed a better performance in the analysis of the undiluted serum than the diluted serum (data not shown). This demonstrates the QCM nanosensor is less sensitive to matrix effects than other analytical techniques [8]. The specificity of the analysis is ensured by monoclonal anti-TnT. Table 1 displays the statistical parameters of the experiments for the detection of TnT in human serum using the nanostructured immunosensor.

The immunosensor exhibited a range from 0.1 ng mL^−1^ to 0.5 ng mL^−1^ (Y = 3.85518 – 63.81882X; SD = 1.019), according to the calibration curve in Figure 5. These data were processed using the ORIGIN program version 8.0 (Microsoft, Redmond, WA, USA) and produced high linearity (R = 0.989; p < 0.001; n = 5), combined with a relative error of 6.4%. The coefficient of variation was 7.0%, obtained with human serum with a detection limit of 0.001 ng mL^−1^. The detection limit was calculated as the concentration of analyte giving a frequency shift equivalent to three standard deviations of the blank. The immunosensor exhibited detection limit values comparable with those of ECLIA methods and is also expected to have no limitations concerning sample color or matrix, which is often a problem in ELISA [32]. The nanosensor demonstrated the ability to measure the concentration of TnT in serum, which makes it a useful method for diagnosing myocardial damage in clinical practice.

The World Health Organization establishes the desirable blood level of TnT to be equal to or less than 0.01 ng mL^−1^ for healthy individuals and beginning at 0.3 ng mL^−1^ as the criterion for the diagnosis of myocardial infarction based on this biomarker [1,2]. The immunosensor proposed in the present study proved to be a sensitive method for measuring the concentration of TnT below the cutoff point for the diagnosis of myocardial infarction and is faster than conventional methods for the diagnosis of acute myocardial infarction.

## 4. Conclusions

The QCM immunosensor based on the sulfur-gold nanoparticle-SAM bond developed in the present study demonstrated high sensitivity for recognizing TnT in human serum and is therefore suitable for clinical application. This nanosensor has the advantage of having a fast response and allowing a label-free analysis of serum. Compared with other conventional tests, the detection of TnT through this nanosensor showed the advantage of saving time and cost, in addition to adequate sensitivity to the range of clinical relevance.

## Figures and Tables

**Table 1.** Parameters of analytical features obtained for detection of TnT in human serum.

**Linear range (ng mL^−1^)**	**Slope (Hertz/ng mL^−1^)**	**Intercept**	**Correlation Coefficient (r) (n = 5)**	**LOD (ng mL^−1^)**	**CV (%)**
0.1 to 0.5	63.82	1.836	0.989	0.0015	7.0

## References

[1] Allender S., Scarborough P., Peto V., Rayner M., Leal J., Luengo-Fernandez R., Gray A. (2008). European Cardiovascular Disease Statistics.

[2] Alpert J.S., Thygesen K., Antman E., Bassand J.P. (2000). Myocardial infarction redefined: A consensus document of the joint European society of cardiology/american college of cardiology committee for the redefinition of myocardial infarction. J. Am. Coll. Cardiol.

[3] Anuário Estatístico de Saúde do Brasil 2001. http://portal.saude.gov.br/saude/aplicacoes/anuario2001/index.cfm/.

[4] Wu A.H.B., Apple F.S., Gibler W.B., Jesse R.L., Warshaw M.M., Valdes R. (1999). National academy of clinical biochemistry standards of laboratory practice: Recommendations for the use of cardiac markers in coronary artery diseases. Clin. Chem.

[5] Perry S.V. (1998). Troponin T: Genetics, properties and function. J. Muscle Res. Cell Motil.

[6] Hamm C.W. (2001). The diagnostic role of troponins. Thromb. Res.

[7] Wei B., Jin J.-P. (2011). Troponin T isoforms and posttranscriptional modifications: Evolution, regulation and function. Arch. Biochem. Biophys.

[8] Ohtsuki I., Morimoto S. (2008). Troponin: Regulatory function and disorders. Biochem. Biophys. Res. Commun.

[9] Wu J., Cropek D.M., West A.C., Banta S. (2010). Development of a troponin I biosensor using a peptide obtained through phage display. Anal. Chem.

[10] Yang Z., Zhou D.M. (2006). Cardiac markers and their point-of-care testing for diagnosis of acute myocardial infarction. Clin. Biochem.

[11] Maynard S.J., Mentown I.B.A., Adgey A.A. (2000). Troponin T or troponin I as cardiac markers in ischaemic heart disease. Heart.

[12] Sauerbrey G.Z. (1959). Use of a quartz vibrator for weighing thin layers on a microbalance. Z. Phys.

[13] Thevenot D.R., Toth K., Durst R.A., Wilson G.S. (2001). Electrochemical biosensors: Recommended definitions and classification. Biosens. Bioelectron.

[14] Dutra R.F., Kubota L.T. (2007). An SPR immunosensor for human cardiac troponin T using specific binding avidin to biotin at carboxymethyldextran-modified gold chip. Clin. Chim. Acta.

[15] Wu J., Tang J.H., Dai Z., Yan F., Ju H.X., Murr N.E. (2006). A disposable electrochemical immunosensor for flow injection immunoassay of carcinoembryonic antigen. Biosens. Bioelectron.

[16] Poitras C., Tufenkji N. (2009). A QCM-D-based biosensor for *E. coli* O157:H7 highlighting the relevance of the dissipation slope as a transduction signal. Biosens. Bioelectron.

[17] Schuhmann W. (2002). Amperometric enzyme biosensors based on optimized electron-transfer pathways and non-manual immobilisation procedures. Rev. Mol. Biotech.

[18] Chand R.S., Gupta B.D. (2007). Surface plasmon resonance based fiber-optic sensor for the detection of pesticide. Sens. Actuat. B.

[19] Gan N., Hou J., Hu F., Zheng L., Ni M., Cao Y. (2010). An amperometric immunosensor based on a polyelectrolyte/gold magnetic nanoparticle supramolecular assembly—Modified electrode for the determination of HIV p24 in serum. Molecules.

[20] Frasconi M., Tortolini C., Botre F., Mazzei F. (2010). Multifunctional Au nanoparticle dendrimer-based surface plasmon resonance biosensor and its application for improved insulin detection. Anal. Chem.

[21] O’Sullivan C.K., Guilbault G.G. (1999). Commercial quartz crystal microbalances—Theory and Applications. Biosens. Bioelectron.

[22] Liu Y.C., Wang C.M., Hsiung K.P. (2001). Comparison of different protein immobilization methods on quartz crystal microbalance surface in flow injection immunoassay. Anal. Biochem.

[23] Chu X., Zhao Z.-L., Shen G.-L., Yu R.-Q. (2006). Quartz crystal microbalance immunoassay with dendritic amplification using colloidal gold immunocomplex. Sens. Actuat. B.

[24] Zhang Y., Wang H., Yan B., Zhang Y., Li J., Shen G., Yu R. (2008). A reusable piezoelectric immunosensor using antibody-adsorbed magnetic nanocomposite. J. Immunol. Meth.

[25] Luo X., Morrin A., Killard A.J., Smyth M.R. (2006). Application of nanoparticles in electrochemical sensors and biosensors. Electroanalysis.

[26] Dutra R.F., Mendes R.K., Lins da Silva V., Kubota L.T. (2007). Surface plasmon resonance immunosensor for human cardiac troponin T based on self-assembled monolayer. J. Pharm. Biomed. Anal.

[27] Bunde R.L., Jarvi E.J., Rosentreter J.J. (1998). Piezoelectric quartz crystal biosensors. Talanta.

[28] Jin X., Jin X., Chen L., Jiang J., Shen G., Yu R. (2009). Piezoelectric immunosensor with gold nanoparticles enhanced competitive immunoreaction technique for quantification of aflatoxin B1. Biosens. Bioelectron.

[29] Wu Z.S., Li J.S., Luo M.H., Shen G.L., Yu R.Q. (2005). A novel capacitive immunosensor based on gold colloid monolayers associated with a sol-gel matrix. Anal. Chim. Acta.

[30] Seydack M. (2005). Nanoparticle labels in immunosensing using optical detection methods. Biosens. Bioelectron.

[31] Shi Y.T., Yuan R., Chai Y.Q., Tang M.Y., He X.L. (2007). Amplification of antigen-antibody interactions via back-filling of HRP (Horseradish peroxidase) on the layer-by-layer self-assembling of thionine and gold nanoparticles films on titania nanoparticles/gold nanoparticles-coated Au electrode. J. Electroanal. Chem.

[32] Manso J., Mena M.L., Yánez-Sedeno P., Pingarrón J.M. (2007). Electrochemical biosensors based on colloidal gold-carbon nanotubes composite electrodes. J. Electroanal. Chem.

